# Supplementary material for: Production of Circularly Permuted Caspase-2 for Affinity Fusion-Tag Removal: Cloning, Expression in Escherichia coli, Purification, and Characterization
Source: Biomolecules. 2020 Nov 24;10(12):1592. doi: 10.3390/biom10121592 (PMC7760212; doi:10.3390/biom10121592)

wtCasp2

IMAC Capture

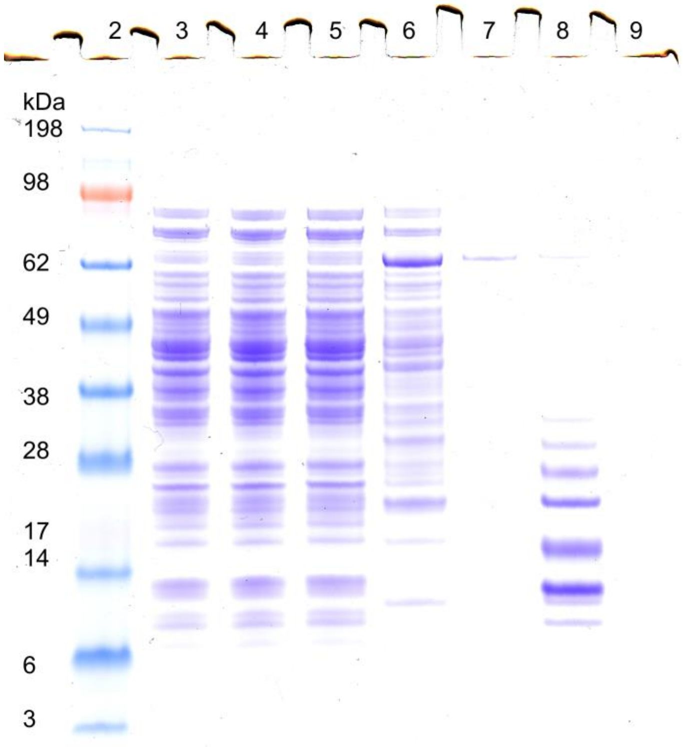

2	Ladder
3	Load
4 & 5	Flow-through
6 & 7	Wash
8	Elution

CIEX Polishing

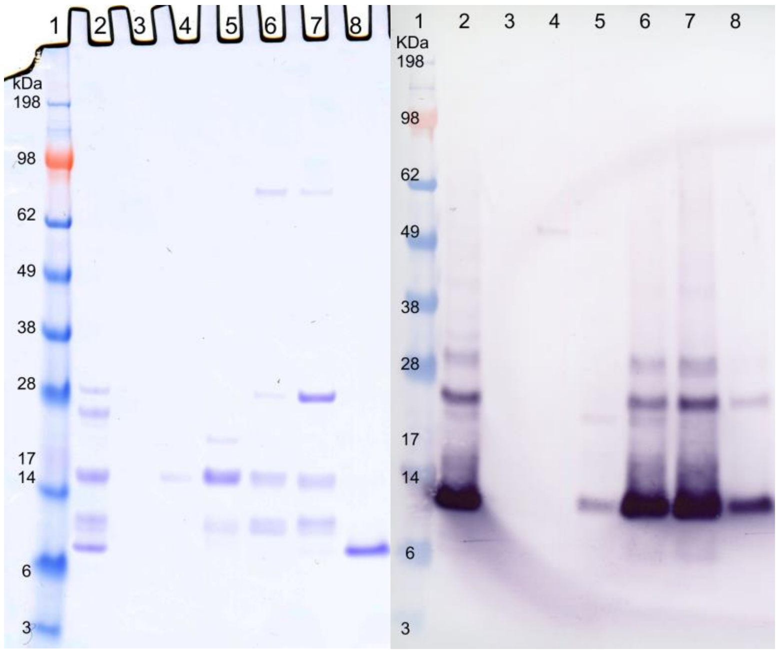

1	Ladder
2	Load
3	Flow-through
4 - 8	Elution

Western blot
anti-Casp2 small subunit AB

cpCasp2 IMAC Capture

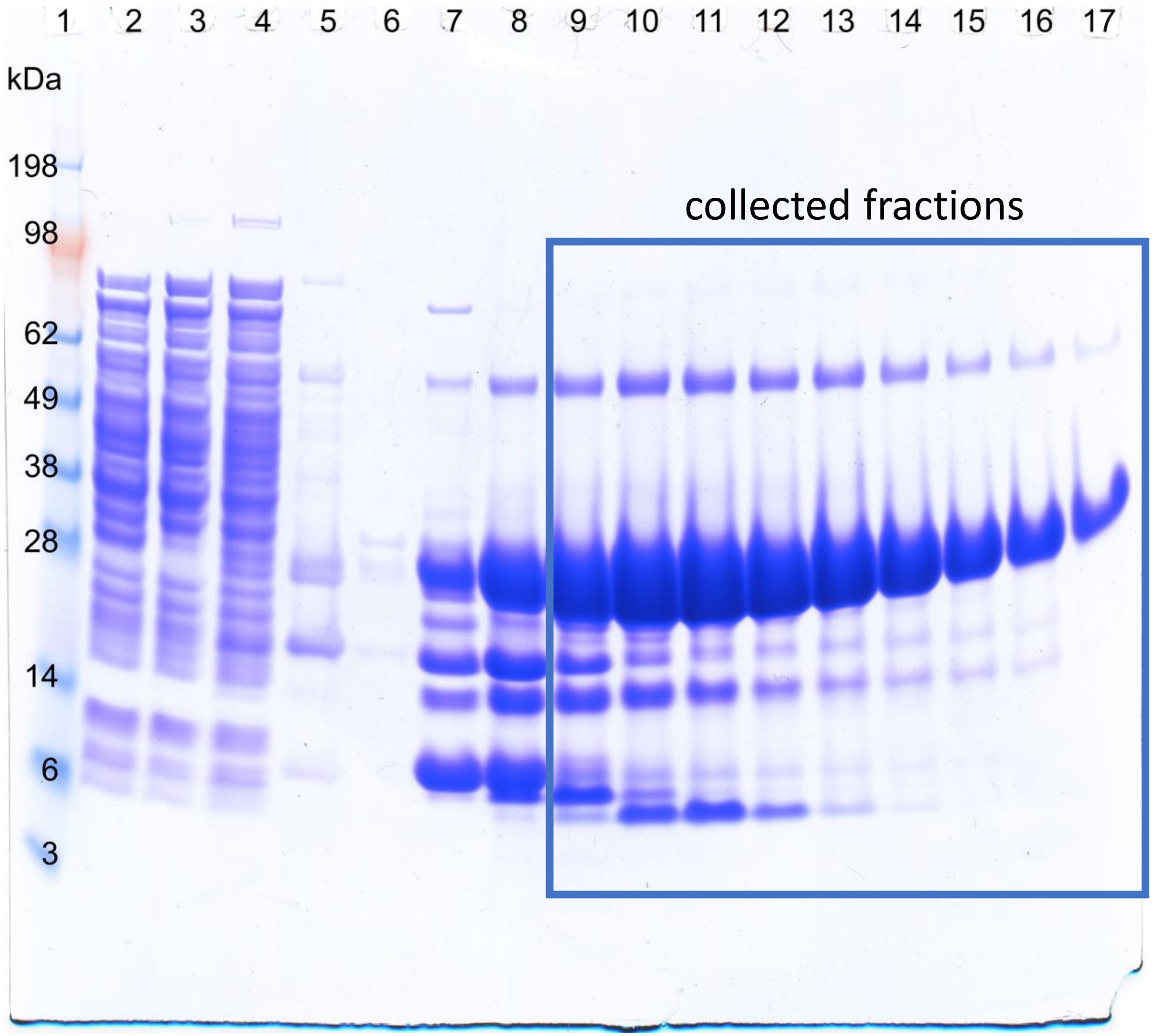

1	Ladder
2	Load 1:5
3	Flowthrough 1:5
5	Wash buffer A
6	Wash isopropanol
7	Wash buffer A
8 - 17	Elution

cpCasp2 CIEX Polishing

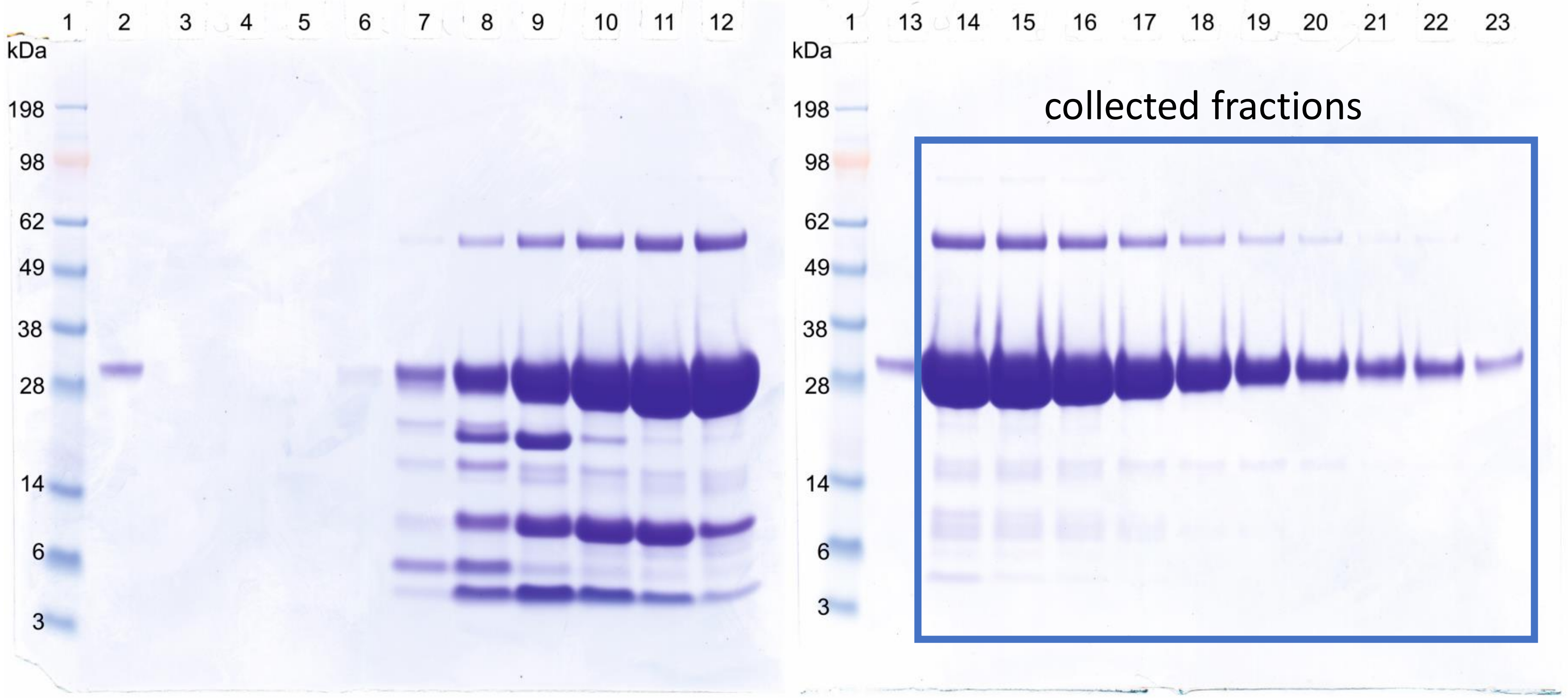

1	Ladder
2 & 13	Load 1:5
3	Flowthrough
4 - 23	Elution

cpCasp2 RP-HPLC purity

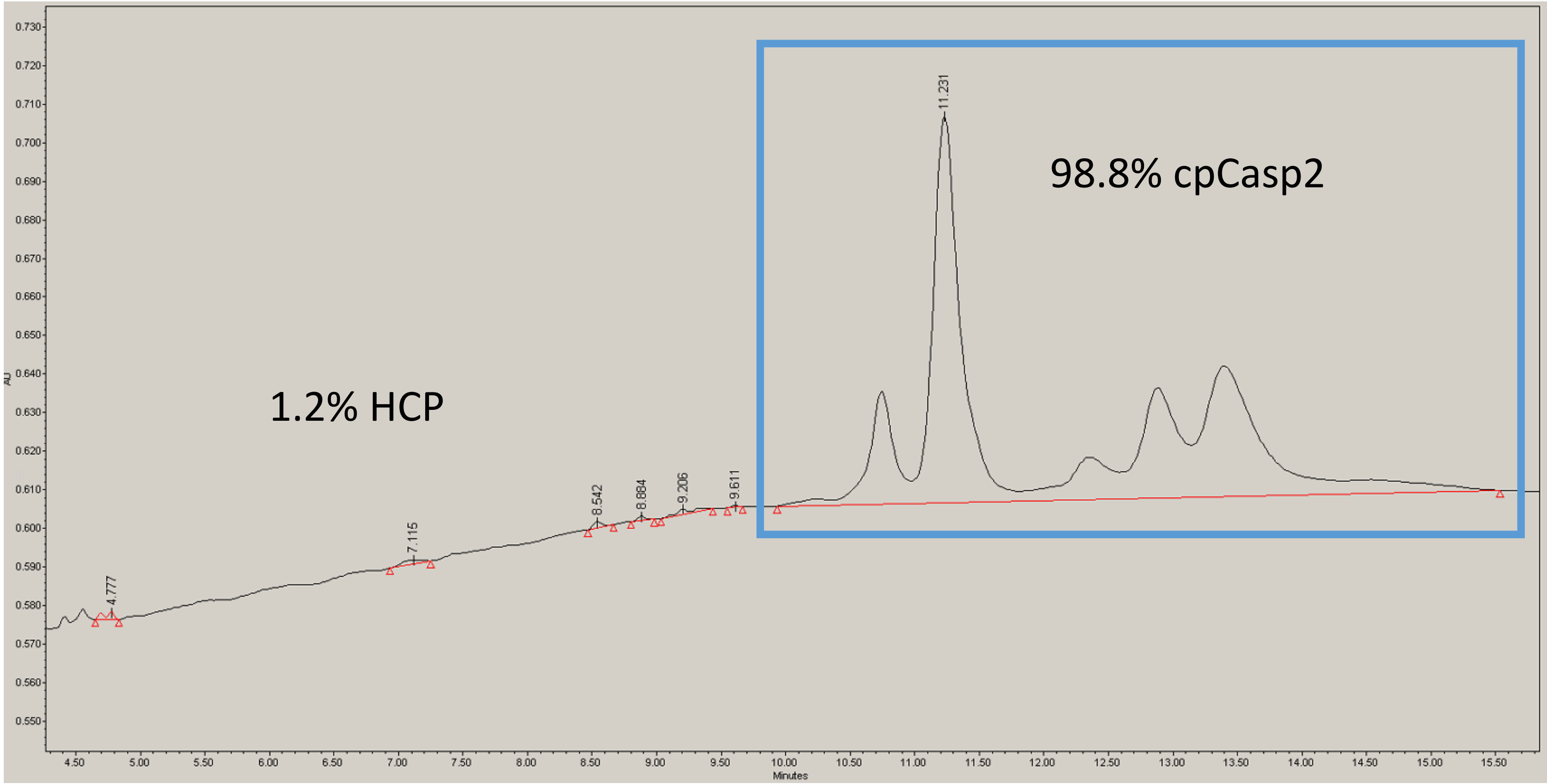

T7AC-cpCasp2 RP-HPLC purity

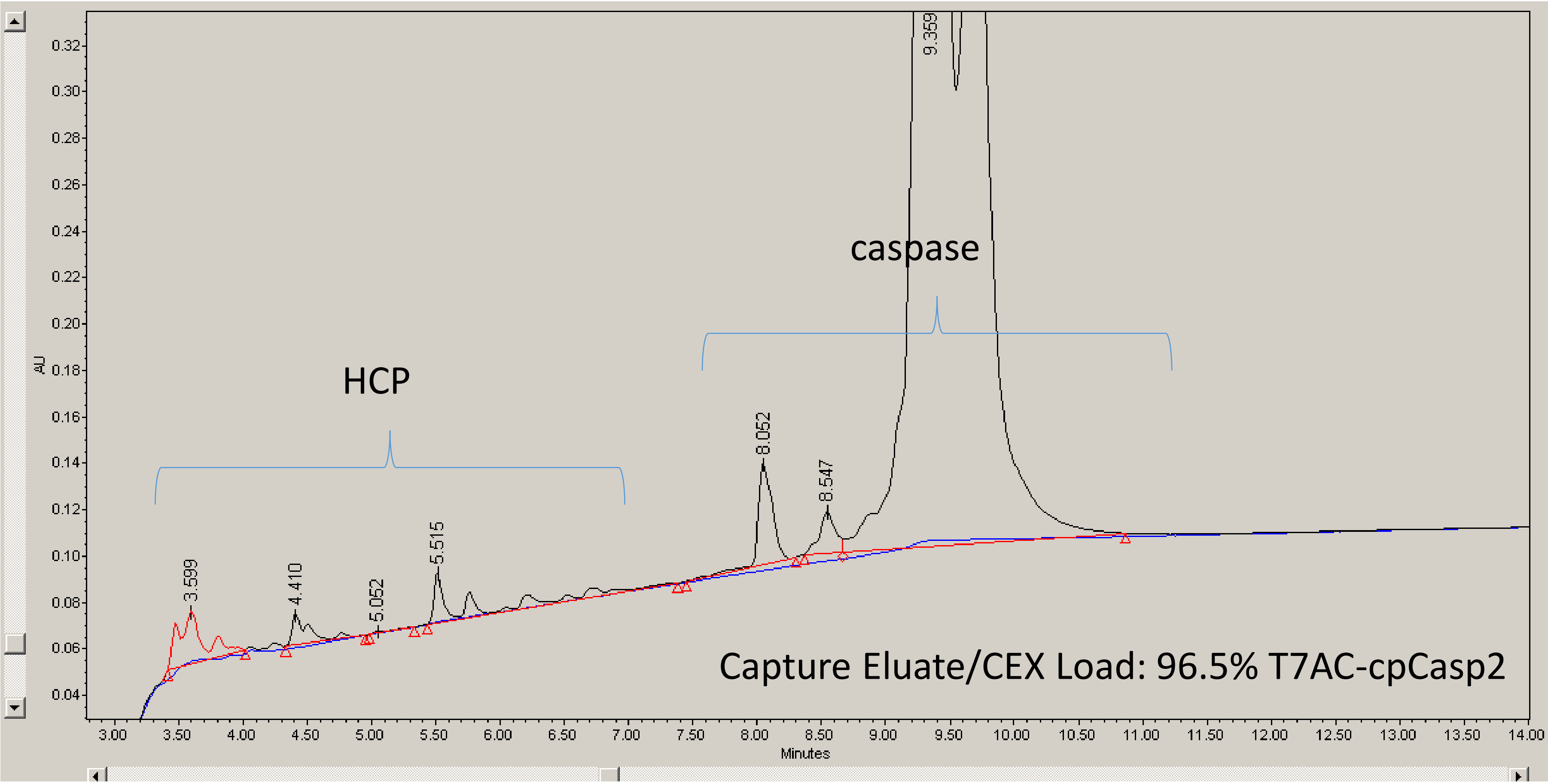

T7AC-cpCasp2 RP-HPLC purity

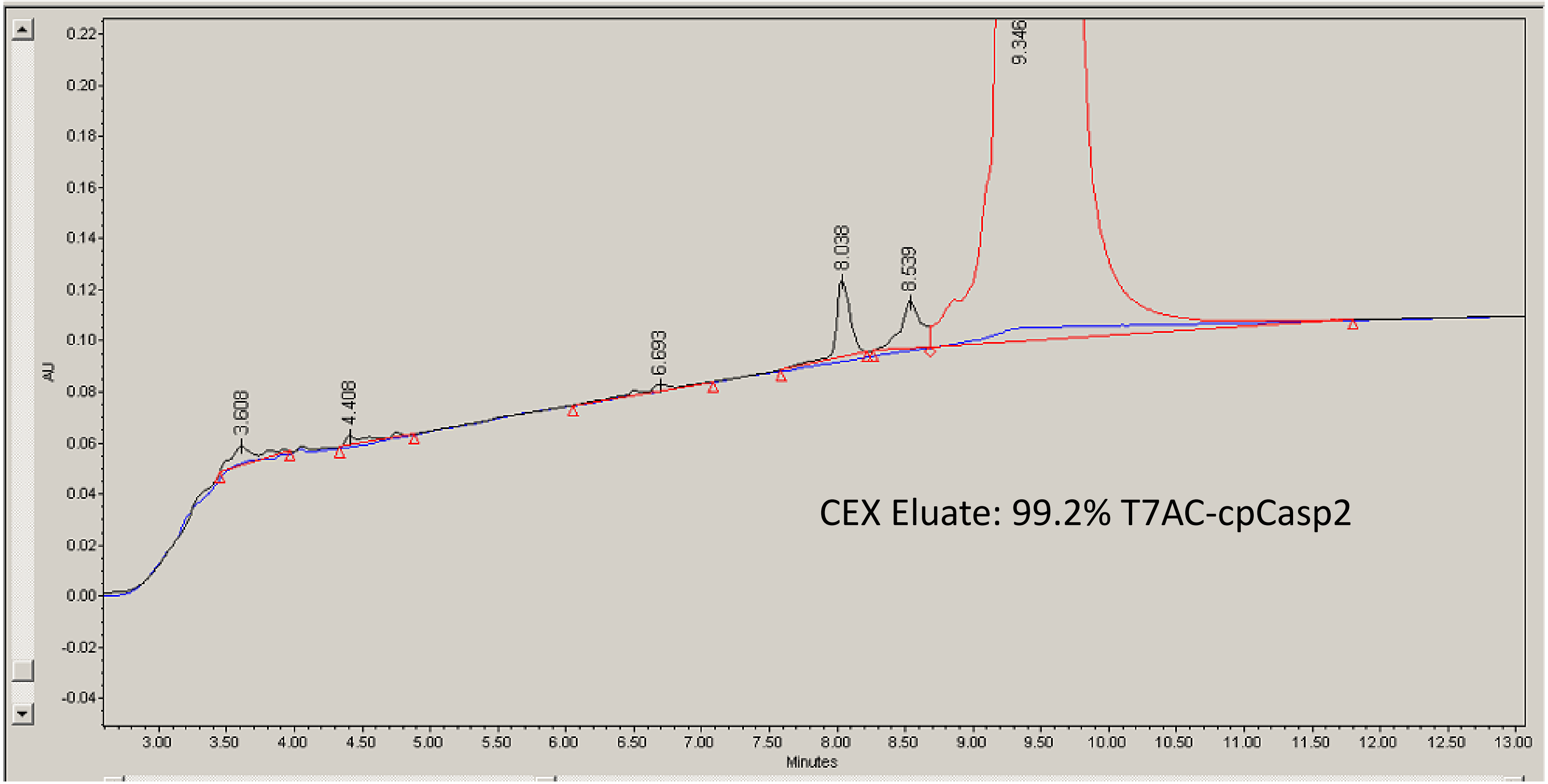

Supplement: Supplementary file 1 [file biomolecules-10-01592-s001.zip › File S4.pdf]
