# Supplementary material for: Production of Circularly Permuted Caspase-2 for Affinity Fusion-Tag Removal: Cloning, Expression in Escherichia coli, Purification, and Characterization
Source: Biomolecules. 2020 Nov 24;10(12):1592. doi: 10.3390/biom10121592 (PMC7760212; doi:10.3390/biom10121592)
Supplement: Supplementary file 1 [file biomolecules-10-01592-s001.zip › File S6.pdf]

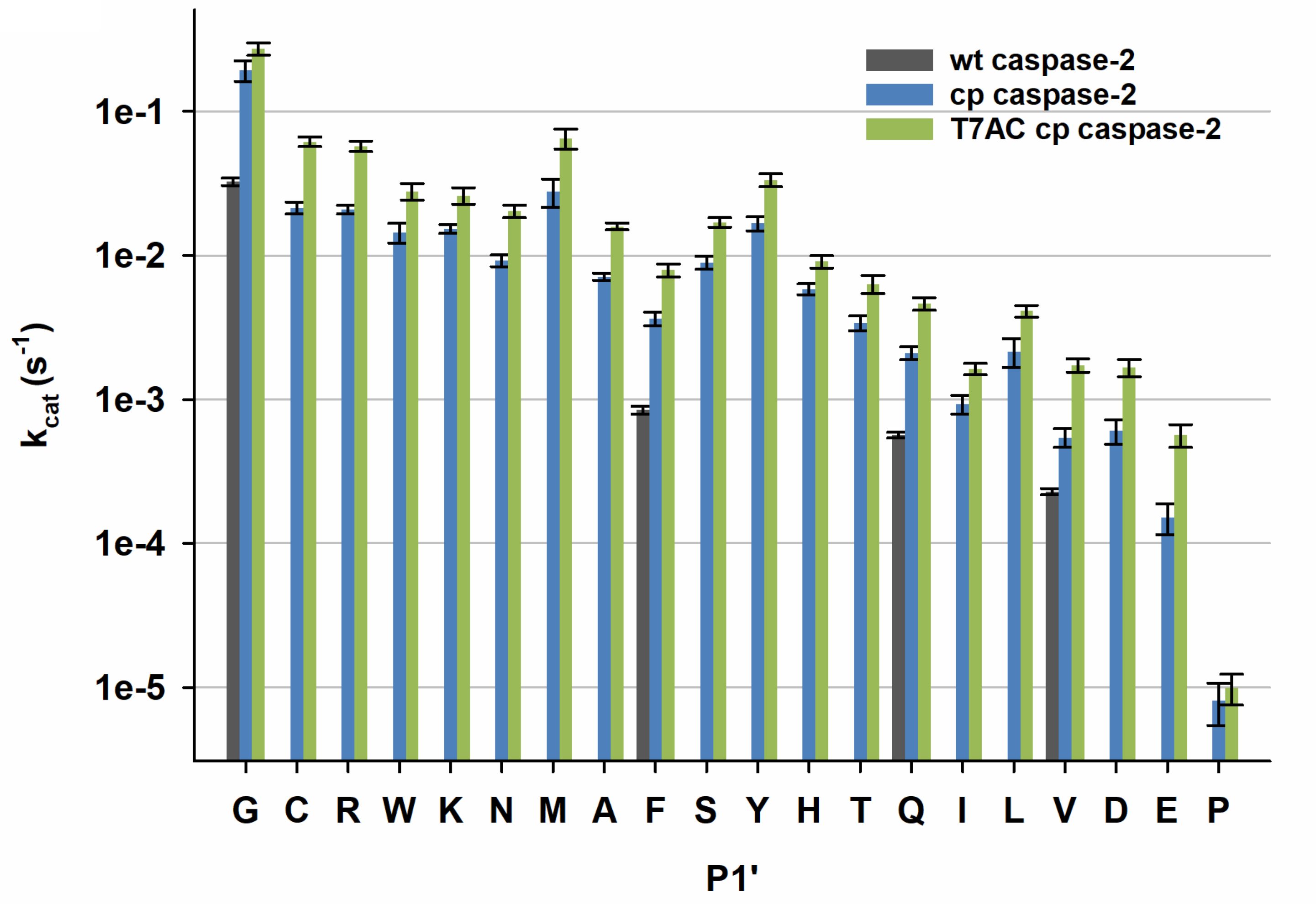

Figure S6. *(a) Enzyme kinetic k_{cat} values determined by FRET assay of wtCasp2, cpCasp2 and T7AC-cpCasp2. Due to the low available amount of wtCasp2 only G, F, Q and V were measured. The error bars denote the 95% confidence interval of the Michaelis Menten model fit (n=15).*

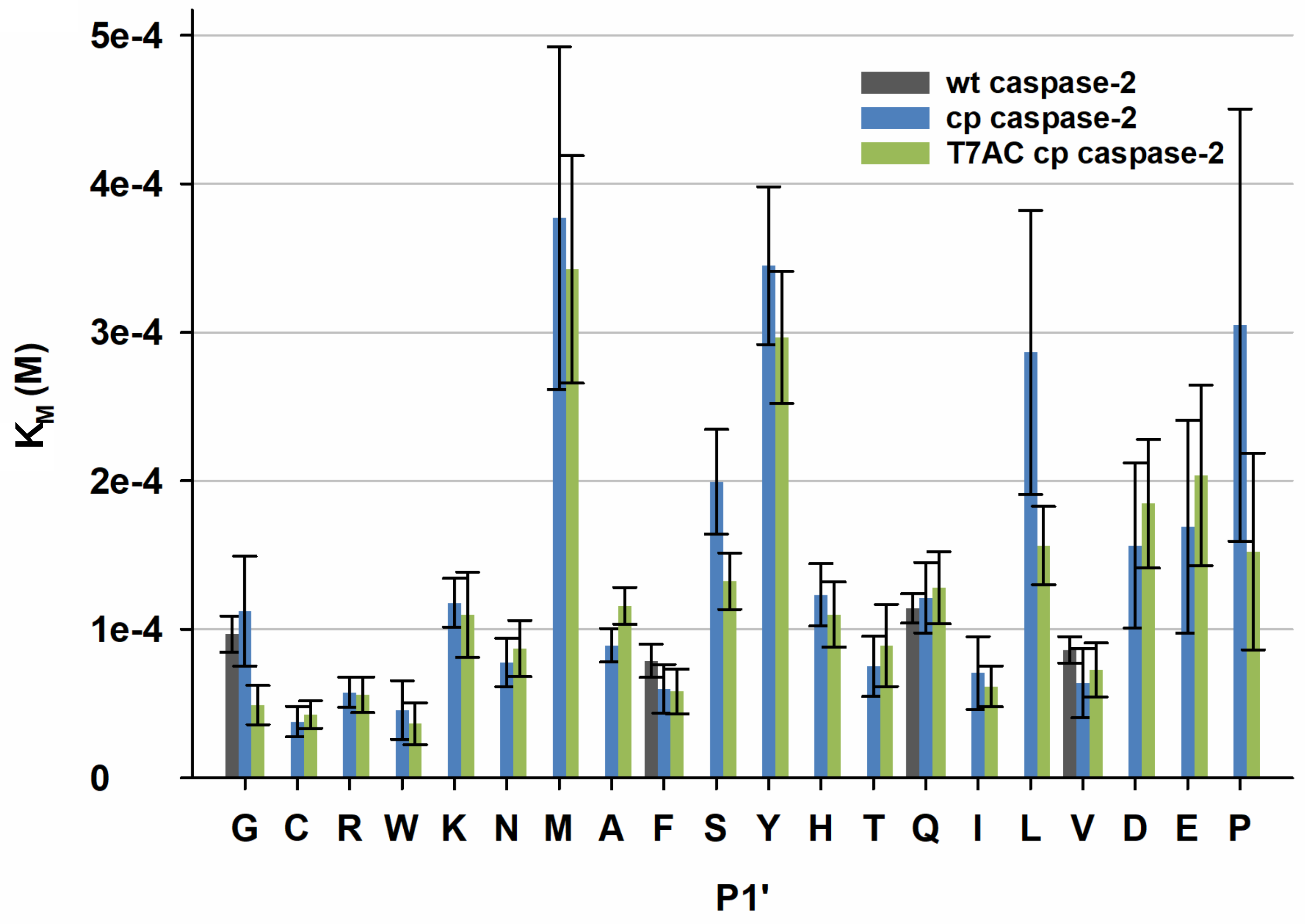

Figure S6. *(a) Enzyme kinetic K_M values determined by FRET assay of wtCasp2, cpCasp2 and T7AC-cpCasp2. Due to the low available amount of wtCasp2 only G, F, Q and V were measured. The error bars denote the 95% confidence interval of the Michaelis Menten model fit (n=15).*
